# MsImpute: Estimation of Missing Peptide Intensity Data in Label-Free Quantitative Mass Spectrometry

**DOI:** 10.1016/j.mcpro.2023.100558

**Published:** 2023-04-25

**Authors:** Soroor Hediyeh-Zadeh, Andrew I. Webb, Melissa J. Davis

**Affiliations:** 1 Bioinformatics Division, WEHI, Melbourne, Australia; 2 Department of Medical Biology, University of Melbourne, Melbourne, Australia; 3 Colonial Foundation Healthy Ageing Centre, WEHI, Melbourne, Australia; 4 Advanced Technology and Biology Division, WEHI, Melbourne, Australia; 5 Department of Clinical Pathology, Faculty of Medicine, Dentistry and Health Sciences, The University of Melbourne, Melbourne, Australia; 6 The Diamantina Institute, The University of Queensland, Brisbane, Australia; 7 The South Australian Immunogenomics Cancer Institute, The University of Adelaide, Adelaide, Australia

**Keywords:** imputation, label-free quantification, missing data, estimation of variance, barycenter computation

## Abstract

Mass spectrometry (MS) enables high-throughput identification and quantification of proteins in complex biological samples and can provide insights into the global function of biological systems. Label-free quantification is cost-effective and suitable for the analysis of human samples. Despite rapid developments in label-free data acquisition workflows, the number of proteins quantified across samples can be limited by technical and biological variability. This variation can result in missing values which can in turn challenge downstream data analysis tasks. General purpose or gene expression-specific imputation algorithms are widely used to improve data completeness. Here, we propose an imputation algorithm designated for label-free MS data that is aware of the type of missingness affecting data. On published datasets acquired by data-dependent and data-independent acquisition workflows with variable degrees of biological complexity, we demonstrate that the proposed missing value estimation procedure by *barycenter computation* competes closely with the state-of-the-art imputation algorithms in differential abundance tasks while outperforming them in the accuracy of variance estimates of the peptide abundance measurements, and better controls the false discovery rate in label-free MS experiments. The *barycenter* estimation procedure is implemented in the *msImpute* software package and is available from the Bioconductor repository.

## Highlights

• *msImpute* provides imputation that is aware of the type of missingness in data
• More-accurate estimates of variance and better control of the false discovery rate
• The *msImpute* software package is available from the Bioconductor repository

## Graphical Abstract

**Figure:**
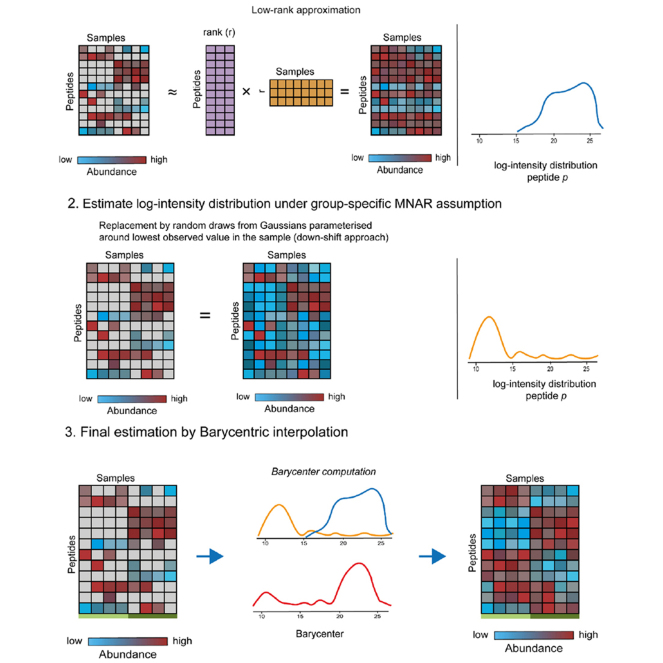

## In Brief

The number of proteins quantified across samples by label-free mass-spectrometry (MS) is limited by technical and biological variability resulting in missing values that challenge downstream analysis. We present an imputation algorithm for label-free MS data that is aware of the type of missingness affecting data. Missing value estimation by msImpute outperforms state-of-the-art imputation methods in the accuracy of variance estimates for peptide abundance and better controls the false discovery rate in MS experiments. msImpute is available from the Bioconductor repository.

Liquid chromatography–coupled tandem mass spectrometry (LC-MS/MS) is the leading technology for quantitative analysis of proteins expressed in samples. Proteins in cell or tissue lysates are first prepared for analysis by extracting the protein content, followed by enzymatic digestion, converting them into peptides. Peptides are separated using LC which is interfaced with the source of the mass spectrometer, where they are ionized and converted to the gas phase. The separated and ionized peptide precursors are subjected to mass analysis in a mass spectrometer. During conventional data-dependent acquisition (DDA), peptide ions are sampled for fragmentation and identified from the spectra produced by tandem mass (MS/MS) analysis using peptide identification software (1). The mass spectrometer, however, only selects a small subset (usually 10) of the most abundant peptides for sequencing by MS/MS at each MS1 survey scan in a run. This impedes consistent identification of peptides across runs, as the sets of peptide precursor ions selected for sequencing could differ between runs. The low sampling efficiency and stochastic nature of intensity-dependent sampling of peptide ions for MS/MS analysis limits the number of peptides and proteins common to all runs and hinders quantification of low abundance ions in complex samples, which contributes to the pervasive occurrence of missing values. Alternative data acquisition workflows such as data-independent acquisition (DIA), which requires prior knowledge about the fragment ion spectra of targeted peptides, have substantially enhanced the reproducibility of proteome quantification across runs and reduced the prevalence of missing values (2). However, the broad dynamic range of proteome quantification in DDA acquisition and the ability to identify peptide sequences from spectral searching makes it the preferred method of choice for label-free quantification in discovery-based proteomics studies, and so missingness remains a problem that is important to address.

Missing values are generally classified into three categories: Missing Completely At Random (MCAR), Missing At Random (MAR), and Missing Not At Random (MNAR) (3). MCAR missing values in proteomics data can originate from random errors or stochastic fluctuations during the experimental process. Several different factors are reported to impact accuracy and reproducibility, including sample preparation, sample processing, peptide separation, changes in sample complexity, matrix effects and ion suppression, detector saturation, and other technical factors (4, 5, 6, 7). MAR describes a situation where the possibility of a variable being missing is dependent on other observed variables. In contrast, MNAR is defined as the possibility of a variable being missing is dependent on unobserved variables (3, 8). MAR data in proteomics are produced during data preprocessing, for example, by inaccurate peak detection and deconvolution of co-eluting compounds. MCAR and MAR missing values are both intensity-independent and can be difficult to distinguish (9, 10), while MNAR missing values are considered intensity-dependent (9, 10, 11). Furthermore, MNAR missing values can occur in a group-specific manner due to the downregulation of a protein in a treatment arm or real variation in the biology of samples in different groups (10). It has been reported that proteomics data often contain a mixture of MAR and MNAR missing values, with the exact MAR/MNAR ratio and composition of missing values difficult to determine in a given dataset (9, 10).

A common approach to increase data completeness is to replace the missing intensity measurements of peptides and proteins that are not quantified commonly across LC-MS/MS runs with some reasonable values by imputation. Imputation methods in proteomics are broadly categorized as left-censored, local similarity, and global similarity approaches (12). Imputation of left-censored MNAR missing values is typically performed by replacing the missing value with the smallest observed value in the run, a random draw from a Gaussian distribution parameterized around such value, or with zero. More sophisticated methods such as quantile regression imputation (QRILC) have also been applied to left-censored data (13). MAR/MCAR is generally difficult to distinguish and can be imputed by local methods based on observed values in the neighborhood using k-nearest neighbors (KNN) (14) or global similarity approaches such as Expectation Maximization (15), Random Forest (RF) (16), Bayesian Principal Component Analysis (BPCA) (17), sequential imputation (18), or multiple imputations by chained equations (19). In contrast to imputation with fixed values, there are also methods that model missing values in an intensity-dependent probabilistic manner (20) to test for differential abundance instead of imputation. While the arguments for this approach focus on the valid and reasonable drawbacks of imputation, such strategies are designed specifically for differential abundance testing. There are many analysis tasks such as clustering, classification, pathway enrichment, and network analysis, that still benefit from accurate imputation procedures.

Previous studies have demonstrated that depending on the composition of missing values in the DDA and DIA mode, several imputation methods or a combination of MAR/MNAR imputation algorithms may be needed (9, 10, 21), in part because existing imputation algorithms were not specifically developed for MS data and are not aware of the compositional nature of missing values in proteomics datasets. In this work, we present an imputation procedure that can adapt to the MAR/MNAR composition of missing values in the proteomics data and demonstrate its performance in the context of differential abundance and empirical false discovery rate (FDR) control.

## Experimental Procedures

### Datasets

Publicly available data containing controlled mixtures or Universal Protein Standards (UPS) spiked-in, where the differentially abundant proteins are known, acquired and analyzed by label-free DDA or DIA were used to benchmark msImpute against state-of-the-art imputation algorithms. A publicly available dataset containing HeLa cell lysate replicates in which no biological variability is present was also used to benchmark empirical FDR control. We have referred to these datasets by the acquisition mode and study throughout this article. Here we describe each dataset.

**DDA:Bruderer2015** (22) is a controlled mixture dataset with 12 proteins spiked into a constant human background in different concentrations generating samples from eight groups (n = 3 replicates) resulting in 24 LC-MS runs. The runs were acquired on a Q Exactive mass spectrometer. The DDA dataset was used. Details of sample preparation and data generation can be found in the original publication. DDA data were analyzed by MaxQuant (1, 23). We used the published *evidence.txt* results. The evidence table was then processed as follows: We only retained the feature with the highest intensity, if multiple ions were reported for a peptide in the evidence table. Contaminants and Reverse Complement identifications were discarded. Peptide intensities were log2 transformed, imputed, and normalized by Quantile Normalization. For imputation using the Accelerated Time Failure model in MSstats (24), we followed the MSstats workflow on Bioconductor for obtaining the abundance matrix. We tested for differential abundance using linear models with Empirical Bayes moderated t-statistics implemented in limma (25, 26). Differential abundance was called if the FDR for a peptide was <0.05. The FDR was computed by the Benjamini–Hochberg procedure. We compared the *Sample8* group *versus Sample1* group. The accession number for this dataset is PASS00589.

**DIA:Huang2020** is a controlled mixture dataset with biological background variation (27). Tissue lysates from 25 mouse cerebellum samples were prepared and five samples (n = 5) were generated in which the UPS2 proteins were spiked in known concentrations, resulting in 25 LC-MS runs in total. The spike-in concentrations were S1: 0.75 amol/μl, S2: 0.83 amol/μl, S3: 1.07 amol/μl, S4: 2.04 amol/μl, and S5: 7.54 amol/μl. The runs were acquired on a Q Exactive HF mass spectrometer followed by the DIA method. Details of sample preparation and data generation can be found in the original publication. DIA data were analyzed with Spectronaut Pulsar X (28). We used the published Spectronaut results in Spike-in-biol-var-OT-SN-Report.txt. Peptide intensities were log2 transformed, imputed, and normalized by Quantile Normalization. We tested for differential abundance using linear models with Empirical Bayes moderated t-statistics implemented in limma (25, 26). Differential abundance was called if the FDR for a peptide was <0.05. The FDR was computed by the Benjamini–Hochberg procedure. We compared the S5 group *versus S1* group. The accession number for this dataset is PXD016647.

**DDA:Giai Gianetto** contains Label-free quantification of various concentrations of Universal Proteomic Standard (UPS1, Sigma-Aldrich) spiked in yeast extract (29). Three concentrations of UPS1 (25 fmol, 10 fmol and 5 fmol) were spiked in yeast extract generating two samples (n = 3) where UPS1 was spiked in 2:1 ratio, and another set of two samples (n = 3) where the UPS1 proteins were spiked in 2.5:1 ratio. The LC-MS runs were acquired on a Q Exactive mass spectrometer. Details of sample preparation and data generation can be found in the original publication. DDA data were analyzed by MaxQuant. We used the published evidence.txt results for these two datasets (that is ratio 2 and ratio 2.5). The evidence table was then processed as follows: We only retained the feature with the highest intensity, if multiple ions were reported for a peptide in the evidence table. Contaminants and Reverse Complement identifications were discarded. Peptide intensities were log2 transformed, imputed, and normalized by Quantile Normalization. For imputation using the Accelerated Time Failure model in MSstats (24), we followed the MSstats workflow for obtaining the abundance matrix. We tested for differential abundance using linear models with Empirical Bayes moderated t-statistics implemented in limma (25, 26). Differential abundance was called if the FDR for a peptide was less than 0.05. The FDR was computed by the Benjamini–Hochberg procedure. We compared the D group *versus* C group in DDA:Giai Gianetto-Ratio 2.5, and the E group to D group in DDA:Giai Gianetto. The accession number for this dataset is PXD002370.

**DDA:Choi2017** is the IPRG-2015 Study (30) in which a set of proteins were spiked in different concentrations in four samples (n = 3) resulting in 12 LC-MS runs. The runs were acquired on a Q Exactive mass spectrometer. Details of sample preparation and data generation can be found in the original publication. DDA data were analyzed by MaxQuant. We used the published evidence.tsv results from this study (retrieved from MSV000079843). The evidence table was then processed as follows: We only retained the feature with the highest intensity, if multiple ions were reported for a peptide in the evidence table. Contaminants and Reverse Complement identifications. Peptide intensities were log2 transformed, imputed, and normalized by Quantile Normalization. For imputation using the Accelerated Time Failure model in MSstats (24), we retrieved the input data for this study from the MassIVE.quant (31) resource (MSV000079843) and followed the MSstats workflow for obtaining the abundance matrix. We tested for differential abundance using linear models with Empirical Bayes moderated t-statistics implemented in limma (25, 26). Differential abundance was called if the FDR for a peptide was <0.05. The FDR was computed by the Benjamini–Hochberg procedure. We compared *sample4* group *versus sample2* group. The accession number for this dataset is PXD015300.

**DDA:Cox2014** (32) is the dynamic range benchmark dataset which generated one sample (n = 4) with UPS1 standards and one sample (n = 4) with UPS2 standards spiked into *Escherichia coli* lysates, resulting in eight LC-MS runs overall. Details of sample preparation and data generation can be found in the original publication. DDA data were analyzed by MaxQuant. We used the evidence.txt results. The evidence table was then processed as follows: we only retained the feature with the highest intensity, if multiple ions were reported for a peptide in the evidence table. Contaminants and Reverse Complement identifications were discarded. Peptide intensities were log2 transformed, imputed, and normalized by Quantile Normalization. For imputation using the Accelerated Time Failure model in MSstats (24), we retrieved the input data for this study from the MassIVE.quant (31) resource (MSV000081831) and followed the MSstats workflow for obtaining the peptide precursor abundance matrix. We tested for differential abundance using linear models with Empirical Bayes moderated t-statistics implemented in limma (25, 26). Differential abundance was called if the FDR for a peptide was <0.05. The FDR was computed by the Benjamini–Hochberg procedure. We compared the UPS2 group *versus* UPS1 group. The accession number for this dataset is PXD000279.

**DDA:Chiva2014** is a controlled mixture dataset (33) where 30 commercial proteins were prepared in three different subsets of ten proteins each. Proteins from these subsets were spiked to a *E. coli* background in different proportions to prepare five different mixtures in triplicates. Details of sample preparation and data generation can be found in the original publication. The peptide precursor abundance data for MaxQuant (23), Skyline (34), Proteome Discoverer, and Progenesis processing tools were retrieved from MassIVE.quant (31) resource (MSV000084181). Peptide intensities were log2 transformed, imputed, and normalized by Quantile Normalization. We tested for differential abundance using linear models with Empirical Bayes moderated t-statistics implemented in limma (25, 26). Differential abundance was called if the FDR for a peptide was <0.05. The FDR was computed by the Benjamini–Hochberg procedure. We compared the Condition2 group *versus* Condition3 group. The accession number for this dataset is PXD005642.

#### The Ten HeLa Cell Lysate Replicates

We used the ten HeLa cell lysate replicates published (35) to evaluate the distribution of *p*-values under null distribution. The LC-MS runs were acquired on a timsTOF Pro mass spectrometer on a 2 h gradient. Details of sample preparation and data generation can be found in the original publication. We used the published evidence.txt results, which were accessed through PRIDE accession PXD014777. The evidence table was then processed as follows: we only retained the feature with the highest intensity, if multiple ions were reported for a peptide in the evidence table. Contaminants and Reverse Complement identifications were discarded. We computed the *p*-values for differential abundance using linear models with Empirical Bayes moderated t-statistics implemented in limma (25, 26).

### Imputation Algorithms

We used six state-of-the-art imputation algorithms to benchmark our method: BPCA (17), RF (16), multivariate imputation using chained equations (MICE) (19), Sequential imputation (impSeq) (18), Expectation-Maximization (EM) (15), and KNN (14).

### Differential Abundance Testing and ROC Curves

The ROC curves are computed based on −log_10_*p*-value from the differential abundance tests. This value was set to zero for peptides for which the *p*-values were not estimable (*e.g.*, because of NA fold-change - that is, the peptide intensity was censored in at least one experimental condition - or close to zero variances).

### Using HeLa Cell Lysate Replicates to Assess *p* Value Distribution Under the Null Model

The ten HeLa cell lysate replicates were randomly assigned to two groups (n = 5 for each group). We then computed the *p* values for a differential abundance of the peptides between these two groups using the linear model and Empirical Bayes moderation of limma and assessed the uniformity of the distribution of *p* values.

### The msImpute Model for Flexible Imputation of Label-Free Mass Spectrometry Datasets With Complex MAR/MNAR Compositions

#### Estimation of Data Distribution Under MAR Assumption by Low-Rank Approximation

A high-dimensional matrix $X_{m\times n}$ with m features and n observations can be approximated and reconstructed by a number r≤min(m,n) of a linear combination of its features. This is known as low-rank approximation, as a lower number of features (r) than the original data (m) are used for the reconstruction of X. Founded on softImpute-ALS algorithm (36), msImpute fits a low-rank model to the peptide abundance matrix with missing values and reconstructs the complete data matrix as the product of two low-rank matrices.

Let $X_{m\times n}$ denote the filtered, $\log_{2}$ transformed, normalized peptide intensity data matrix with missing values, where m denotes the number of peptides and n denotes the number of LC-MS runs. Denote the indices of non-missing observations by the set Ω. The softImpute-ALS algorithm combines Nuclear-Norm-regularized matrix approximation and maximum-margin matrix factorization to find two low-rank r≤min(m,n) matrices $A_{m\times r}$ and $B_{n\times r}$, such that the incomplete matrix can be reconstructed by the product of the two matrices, *i.e.*, $X\approx AB^{T}$. The two matrices A and B are found by minimizing the following objective function:

(1) $${minimize}_{A,B}\;\frac{1}{2}∥P_{\Omega }\left(X-AB^{T}\right){∥}_{F}^{2}+\frac{\lambda }{2}\left({∥A∥}_{F}^{2}+{∥B∥}_{F}^{2}\right)$$

where $P_{\Omega }$ is the subset of observed peptide intensities, $∥\cdot {∥}_{F}^{2}$ is the nuclear norm that encourages low-rank solutions, and λ is a shrinkage operator that controls the rank of the matrices being estimated. That is, we find two matrices A and B of lower dimensions (rank) than the measured peptide intensities, X, such that their products approximate X over the observed values with a reasonable accuracy (hence, the difference between X and $\tilde{X}=AB^{T}$ becomes negligible, for observed entries of X). The solutions are found by alternating between two Least Squares problems given in Equations 2 and 3.

The matrix A=UD is initialized by the random matrix $U_{m\times r}$ with orthonormal columns and $=I_{r}$, the identity r×r matrix. Given A, solve for B:

(2) $${minimize}_{B}∥P_{\Omega }\left(X-AB^{T}\right){∥}_{F}^{2}+\lambda {∥B∥}_{F}^{2}\text{,}$$

This is a multiresponse ridge regression with solution:

$${\tilde{B}}^{T}={\left(D^{2}+\lambda I\right)}^{-1}DU^{T}X\text{.}$$

B=VD is reconstructed from Singular Value Decomposition (SVD) of $\tilde{B}D=\tilde{V}{\tilde{D}}^{2}R^{T}$, where $V=\tilde{V}$ and $D=\tilde{D}$. Given B, A is solved by

(3) $${minimize}_{A}∥P_{\Omega }\left(X-AB^{T}\right){∥}_{F}^{2}+\lambda {∥A∥}_{F}^{2}\text{,}$$

which is also a multiresponse ridge regression with solution:

$$\tilde{A}=XVD{\left(D^{2}+\lambda I\right)}^{-1}\text{.}$$

A is then updated by the product of two matrices A=UV, where $U=\tilde{U}$ and $V=\tilde{V}$ are estimated from SVD of $\tilde{A}D=\tilde{U}{\tilde{D}}^{2}R^{T}$. These steps are repeated until the difference between successive estimates of $AB^{T}$ becomes negligible (*i.e.*, the algorithm is converged). The parameter λ controls the rank r of A and B matrices, hence ensures the solution to Equation 1 is low-rank. As λ decreases, the rank of solutions tends to increase. Note that the low-rank models assume data points are Missing At Random (MAR), and observations, that is the LC-MS runs, are independent. LC-MS runs from a single fractionated sample are correlated. This induces a dependency between measurements of the corresponding runs, and therefore, violates the assumptions of the low-rank model. Thus, the model is applicable to data using fractionation, only if the runs or raw files from the fractionated sample are merged.

Although softImpute has in-built algorithms to estimate the rank, in practice we have observed that it underestimates the rank in small data settings, say n = 6 LC-MS runs, as is the case with common mass spectrometry studies. We estimate the rank r as the Effective Rank (37) of the data matrix.

### The Effective Rank

Consider the singular value decomposition X = UDV where U and V are matrices of size m×m and n×n, respectively, and D is a m×n diagonal matrix containing the singular values:

$$\xi_{1}\geq \xi_{2}\geq \cdots \geq \xi_{Q}\geq 0\text{,}$$

where Q=min(m,n). Let $\xi ={\left(\xi_{1},\xi_{2},\cdots ,\xi_{Q}\right)}^{T}$ and define the singular value distribution

$$p_{k}=\frac{\xi_{k}}{{∥\xi ∥}_{1}}\;for\;k=1,2,\cdots ,Q\text{,}$$

where the superscript^T^ denotes the transpose and ${∥\cdot ∥}_{1}$ is the $l_{1}$ norm defined as

$${∥\xi ∥}_{1}=\sum_{k=1}^{Q}\left|\xi_{k}\right|\text{.}$$

The effective rank of intensity data matrix X is defined as

$$\hat{r}=erank\left(X\right)=\exp\left\{H\left(p_{1},p_{2},\cdots ,p_{Q}\right)\right\}\text{,}$$

where $H\left(p_{1},p_{2},\cdots ,p_{Q}\right)$ is the Shannon entropy given by

$$H\left(p_{1},p_{2},\cdots ,p_{Q}\right)=-\sum_{k=1}^{Q}p_{k}\;\log\;p_{k}\text{.}$$

### Estimation of Data Distribution Under Group-Specific MNAR Assumption

Here we assume measurements for a single peptide are missing in one or more conditions (groups), most likely because of low abundance in the experiment. Group-specific missing values can occur due to technical reasons, for example, in experimental batches where each batch of the data is run on separate instruments, days, gradient lengths, and so on. The measurements can also be missing due to biological reasons, that is insufficient ion abundance due to downregulation of a protein in a disease condition or upon a perturbation. Under group-specific MNAR assumption, referred to as MNAR interchangeably in this work, missing values are replaced by random draws from a multivariate normal distribution. The multivariate normal distribution was parameterized by the following (vector) of means μ and standard deviations σ:

$$\mu =x^{-}-\left(s\times \hat{\sigma }\right)$$

$$\sigma =\hat{\sigma }\times w\text{,}$$

where x¯ denotes sample means, $\hat{\sigma }$ denotes sample standard deviations, s and w denote shift and width parameters, respectively. We set s=1.8 and w=0.3. This approach is known as the *down-shift* approach (38).

### Estimation of Missing Values by Barycenter Interpolation

We estimate the missing peptide intensity data in label-free mass spectrometry experiments as the barycenter (weighted average) of two distributions: the distribution of the data under the MAR and the MNAR assumptions. The peptide intensity data distribution under the MAR assumption $X^{MAR}$ is estimated by low-rank approximation, whereas the distribution under MNAR assumption $X^{MNAR}$ is estimated by down-shift approach, both described earlier.

Let $X_{i}^{MAR}$ and $X_{i}^{MNAR}$ denote the (univariate) distribution of log-intensity values for peptide i under MAR and MNAR assumptions, respectively. The Frechet Mean or Barycenter of two probability distributions (39) for peptide i is defined as

$$Bar{\left(\alpha_{i}^{j},X_{i}^{j}\right)}_{j\in \left\{MAR,MNAR\right\}}\in {minimize}_{{X_{i}}^{\prime }}\;E\left({X_{i}}^{\prime }\right)=\sum_{j\in \left\{MAR,MNAR\right\}}\alpha_{i}^{j}D\left({X_{i}}^{\prime },X_{i}^{j}\right)\;for\;i=1,2,\cdots ,m\text{,}$$

where $X_{i}^{\prime }$ is the distribution of log-intensity values for peptide i estimated such that its distance D(⋅,⋅) from $X_{i}^{MAR}$ and $X_{i}^{MNAR}$ distributions is weighted by $\alpha_{i}^{MAR}$ and $\alpha_{i}^{MNAR}$ (note weights sum to 1, *i.e.*, $\alpha_{i}^{MAR}+\alpha_{i}^{MNAR}=1$). Therefore, when D is $l_{2}$ (squared Euclidean) distance, $X_{i}^{\prime }$ is a weighted average of distribution of log-intensity values under MAR and MNAR assumption. The peptide-specific weights $\alpha_{i}$ here act as shrinkage operators: If the missing values for a peptide tend to be missing completely in one or more experimental groups, the $X_{i}^{MNAR}$ is weighted more (*i.e.*, $\alpha_{i}^{MNAR}>\alpha_{i}^{MAR}$) and the final estimates $X_{i}^{\prime }$ are shrunken towards imputation by the *down-shift* approach. However, if the missing value distribution is even across the samples for a peptide, the final estimates $X_{i}^{\prime }$ for the peptide are shrunken towards the low-rank imputed values that is $X_{i}^{MAR}$ (*i.e.*, $\alpha_{i}^{MNAR}<\alpha_{i}^{MAR}$). The judgment of randomness of missing values is based on the Entropy of Mixing (40) metric defined below.

### Entropy of Mixing as a Shrinkage Operator

Let $x_{ik}$ denote the log-intensity for peptide i in LC-MS run k, where i={1,2,⋯,m} and k={1,2,⋯,n}. Let $M_{m\times n}$ be a binary matrix with the same dimensions of X defined as

$$M_{i,k}=\left\{ \begin{array}{l} 1,\text{if}\;x_{ik}\;\text{is}\;\text{not}\;\text{missing}\\ 0,\text{otherwise} \end{array}\right.$$

The evenness or group-specific patterns of missing values are determined by computing the entropy of the M matrix defined above. This is equivalent to the following computation:

$$EBM_{i}=\sum_{g=1}^{G}m_{ig}\;\log\left(m_{ig}\right)\text{.}$$

where g=1,2,⋯,G denotes the experimental condition (group) in the study design (*e.g.*, control and treatment groups), and $m_{ig}$ is the proportion of observed, non-missing values in group g for peptide i. This metric is computed for each peptide individually. Since entropy is a measure of randomness, a finite EBM indicates that missing values are evenly distributed across runs. It, therefore, signals a MAR type. For a peptide missing in a group-specific manner, EBM is not finite, indicating a group-specific MNAR type. The shrinkage is therefore determined as:

$$\alpha_{i}^{MAR}=\left\{ \begin{array}{l} 0.8,\text{if}\;EBM_{i}\;\text{is}\;\text{finite}\\ 0.2,\text{otherwise} \end{array}\right.$$

$$\alpha_{i}^{MNAR}=1-\alpha_{i}^{MAR}$$

Currently, the values for α are fixed and are not estimated by an adaptive procedure.

The final estimates $X_{i}^{\prime }$ for a peptide are, therefore, a barycentric interpolation between distribution of intensity values under MAR and MNAR assumptions, according to the evidence (*i.e.*, the EBM metric) of the type of missingness. Note that we are taking an entropic approach to estimate the probability that a given peptide i is missing at random, or not at random. For peptides with sufficient measurements (number of observed values), optimal solutions are always found in the low-rank model. Peptides for which an optimal solution cannot be found must be discarded from the input data.

## Results

### The msImpute Model for Imputation of Missing Values

**Fig. 1:**
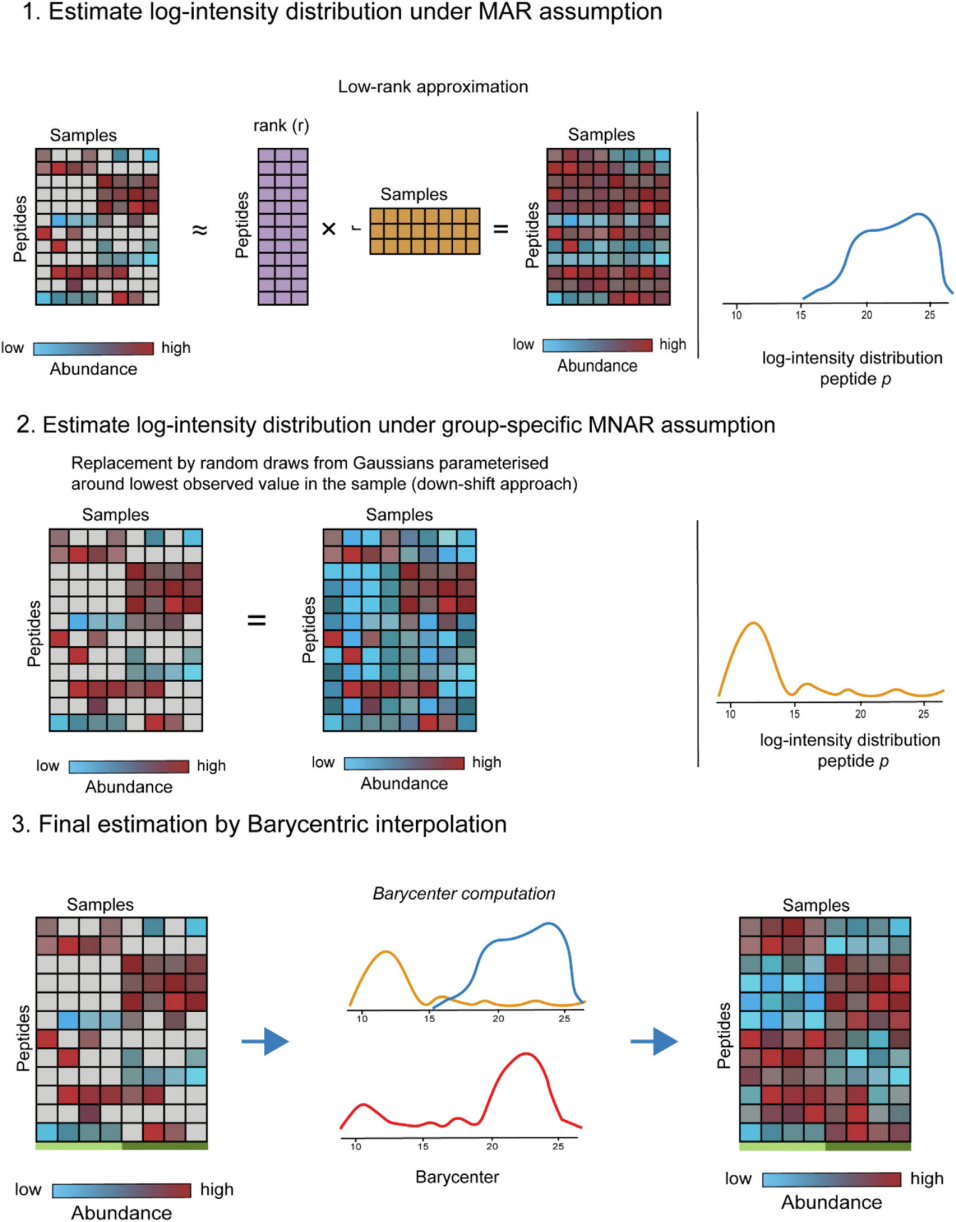
**Schematic illustration of msImpute imputation procedure.** The distribution of intensity values for a single peptide under MAR and group-specific MNAR assumptions are estimated by low-rank approximation (step 1) and down-shift approach (step 2), respectively. An entropic metric is used to weigh these two distributions in a weighted mean. The final estimated distribution of intensity values is a weighted mean, the barycenter, of data under MAR and MNAR assumptions (step 3). The barycenter approach takes into account the compositional nature of MAR/MNAR missing values in proteomics datasets and adopts a MAR- or MNAR-suited imputation method based on the evidence in the data.

The msImpute model (Fig. 1) assumes that each missing peptide intensity occurs with some probability *p* at random (MAR), and therefore, with probability 1 − *p* the occurrence is not at random (NMAR) in a group-specific manner. The likeliness of each of the MAR and MNAR assumptions is determined from the data by an entropy-based metric.

The model reconstructs the distribution of the missing peptide across the LC-MS runs under the MAR and group-specific MNAR assumptions using Low-Rank approximation and the conventional down-shift approach, respectively. It then computes the weighted mean, *i.e.*, the barycenter, of the two re-constructed distributions, where the weight is determined by the entropic metric. The entropic metric serves as evidence for the occurrence of each MAR and group-specific MNAR types.

### Comparison of Differential Abundance Results in Six Controlled Mixture and UPS1/2 Spike-in Datasets

**Fig. 2:**
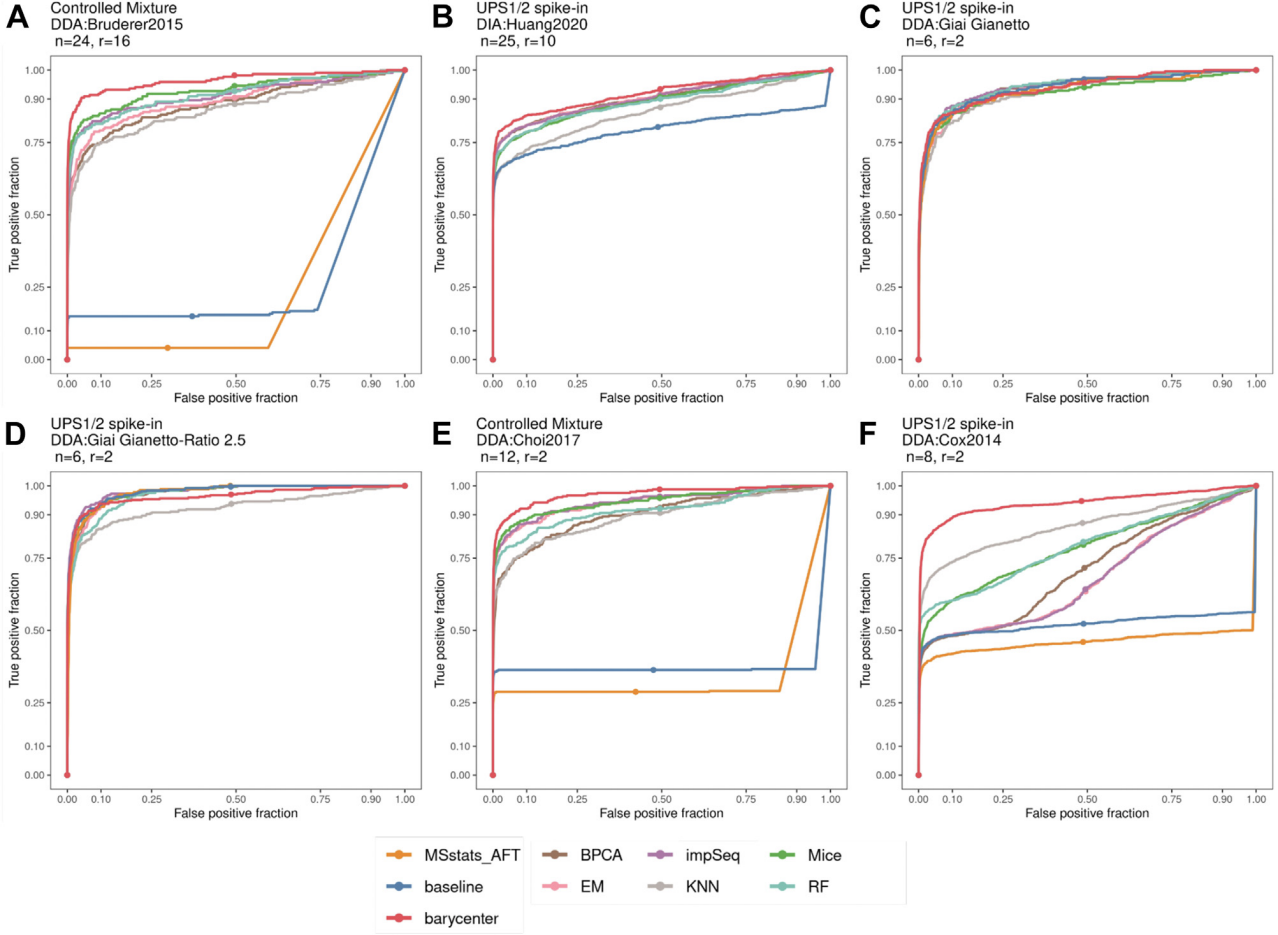
**Differential abundance ROC curves comparing eight imputation procedures to the baseline in published spiked-in, controlled mixture DDA and DIA datasets.** The barycenter (red) estimation procedure is compared to baseline (no imputation) and seven state-of-the-art imputation algorithms in six published label-free datasets with UPS1/2 or exogenous spiked-in proteins. True Positive fraction (x-axis), differentially abundant peptides originated from spiked proteins, and False-Positive fraction (y-axis), peptides not belonging to spiked proteins determined to be differentially abundant at 5% FDR by linear models and Empirical Bayes moderated t-statistics in *limma*. The larger the area under the curve the better. Also shown in panels A-F are each study name, acquisition type, number of LC-MS runs in the study (n) and the rank (r) of the Low-Rank model fitted by msImpute.

We evaluated the number of peptides that were correctly called differentially abundant (True positives), or were falsely detected to be different between the experimental groups (False positives) after imputation in controlled mixtures with constant background (Fig. 2, *A* and *E*), variable background (Fig. 2*B*), or in datasets spiked with UPS1/2 standards (Fig. 2, *C*, *D* and *F*). The number of LC-MS runs in these studies is variable, with some datasets containing as few as six and others as many as 25 runs. Figure 2 shows the fraction of false positive peptides found for each true positive (*i.e.*, correct) differential abundance (DA) calls for different imputation algorithms compared to baseline, that is no imputation at nominal 5% FDR. The area under the curves indicates the sensitivity and specificity of the DA call under each imputation scheme. Therefore, the larger the length of a curve, the better the overall DA outcomes were in the corresponding imputation algorithm. A larger area under the curve suggests that more true positives are found by the imputation procedure. In addition, the respective imputation algorithm would have a better false discovery or false positive control rate.

**Fig. 3:**
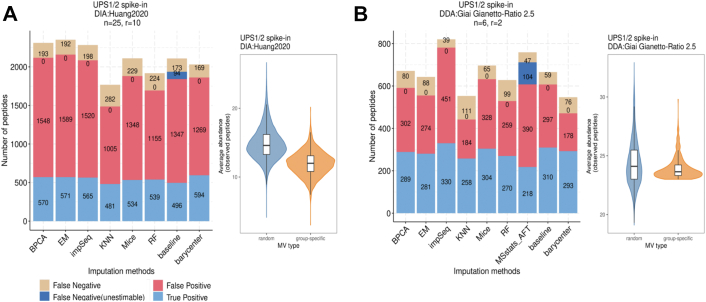
**Data-driven estimates of missing-values type composition determine the reliability of differential abundance test results in barycenter-imputed data.** True positive, false-positive, and false-negative peptide hits in the differential abundance results and violin plot of the mean abundance of peptides exhibiting random (*blue*) and group-specific (*orange*) missing types in datasets where the barycenter approach exhibits an optimal (*A*) and suboptimal (*B*) performance, respectively. The mean abundance is determined based on observed measurements before imputation. The type is determined by the EBM metric. The “unestimable” false-negative peptides indicate that the fold-change could not be determined (that is NA fold-change) because the values were censored (not imputed) in one experimental group. MV, Missing Value.

In DDA- and DIA-controlled mixture datasets with constant and heterogeneous biological backgrounds by Bruderer *et al.* (22) (Fig. 2*A*), Huang *et al.* (27) (Fig. 2*B*), Choi *et al.* (30) (Fig. 2*E*), and Cox *et al.* (32) (Fig. 2*F*), we observed that the barycenter approach maintained the largest area under the curve. We found that the ROC curves for imputation by Accelerated Failure Time model in MSstats (24) (MSstats_AFT), which we could not generate for the DIA:Huang2020 dataset due to software incompatibility problems, and the baseline were indicative of peptides from spiked proteins that were censored (not imputed) in one experimental condition under the test, resulting in unestimable (NA) fold-change and *p*-values. Specifically, our approach resulted in the largest true positives in DIA:Huang2020 (594/763 TP peptides) and in DDA:Cox2014 (856/1121 TP peptides) (Fig. 2*A* and supplemental Fig. S1*B*). We detected lower false positives compared to impSeq (392 by ours *versus* 3607 by impSeq) MICE (392 by ours *versus* 1927 False-positive peptides by MICE) in DDA:Bruderer2015 (supplemental Fig. S1*A*). In the UPS1/2 spike-in dataset DIA:Huang2020, the larger area under the curve for the barycenter approach was the result of largest True positive peptides (594) and lowest False-Negative peptides (169) compared to all other imputation methods (Fig. 3*A*). In addition, in the dynamic benchmark dataset of Cox *et al.* (DDA:Cox2014) we found that more than 60% of the DA calls unique to barycenter were group-specific (supplemental Fig. S2*A*), suggesting that the barycenter approach can detect MNAR peptides that are not detected by other imputation methods.

In the UPS1 spike-in datasets by Gianetto *et al.* (29) (DDA:Giai Gianetto and DDA:Giai Gianetto-Ratio 2.5, see Fig. 2, *C* and *D*), where UPS1 standards were spiked in a yeast background, the barycenter approach recovered a smaller number of peptides from spiked proteins compared to impSeq, but made lower false positive peptide calls (57 FP peptides by ours *versus* 109 by impSeq in DDA:Giai Gianetto, and 178 FP by ours *versus* 451 by impSeq in DDA:Giai Gianetto - Ratio 2.5). We additionally observed that the barycenter approach resulted in lower False Negatives compared to impSeq in these datasets (Fig. 3*B* and supplemental Fig. S1*C*). Since in both datasets by Gianetto *et al.* the baseline (*i.e.*, no imputation) performs as well as imputation, we speculated that the majority of missing peptides are probably of MAR type. We, therefore, sought to validate this by investigating DA outcomes, that is, the number of TP, FP, and FN peptides, for different values of the α, which controls how the MAR (and therefore MNAR) imputed distribution is weighted in the barycenter (supplemental Fig. S2*B*). We found that the smallest FP was achieved for the largest value of alpha, that is where the MAR distribution was highly weighted, although this was also associated with a moderate decrease in TP DA calls and no dramatic performance drops were observed.

In the spike-in datasets by Choi *et al.* (30) (DDA:Choi2017), we found that the proportion of True Positives in barycentric imputation was comparable to Random Forest and impSeq (supplemental Fig. S1*D*), and resulted in lower False Positives compared to Mice (44 ours *versus* 132 in Mice). Furthermore, while 71% of the peptides from the spiked proteins were not imputed and therefore could not be recovered by MSstats AFT imputation model, the barycentric approach recovered 66% of the peptides which are known to change between the mixtures.

We sought to investigate the potential explanations for sub-optimal performance of msImpute-barycenter in Gianetto *et al.* datasets (DDA:Giai Gianetto and DDA:Giai Gianetto-Ratio 2.5) by examining the MAR/MNAR composition in all datasets using the EBM metric. We determined the type of missing values for every peptide as *random* if it had a finite, strictly positive EBM value, or as *group-specific* otherwise (see Experimental Procedures). We then looked at the distribution of the mean abundance of the observed peptides per missing-value type. In the DIA UPS1/2 spike-in dataset DIA:Huang2020, where barycenter imputation exhibited optimal performance in the differential abundance analysis, we observed that the distribution of average abundance of the peptides exhibiting group-specific and random missing-value types were clearly distinguishable (Fig. 3*A*), and the medians of the distributions were clearly shifted. However, in DDA:Giai Gianetto-Ratio 2.5, which is a DDA UPS1/2 spike-in benchmark dataset and where the barycenter imputation had sub-optimal performance relative to baseline, we observed that the median of the distribution of the average abundance of the observed peptides is very close for the peptides exhibiting group-specific and random missing-values types (Fig. 3*B*). The observation that the barycentric imputation ensures optimal or near-optimal performance when the distribution of group-specific and random missing-values types are discernible was also replicable in other benchmark datasets (supplemental Fig. S1, *E*–*H*, see also supplemental Fig. S3). These findings overall suggest that imputation by barycenter computation can have optimal performance as good or better than the state-of-the-art, general-purpose imputation algorithms in real datasets with various MAR/MNAR compositions.

**Fig. 4:**
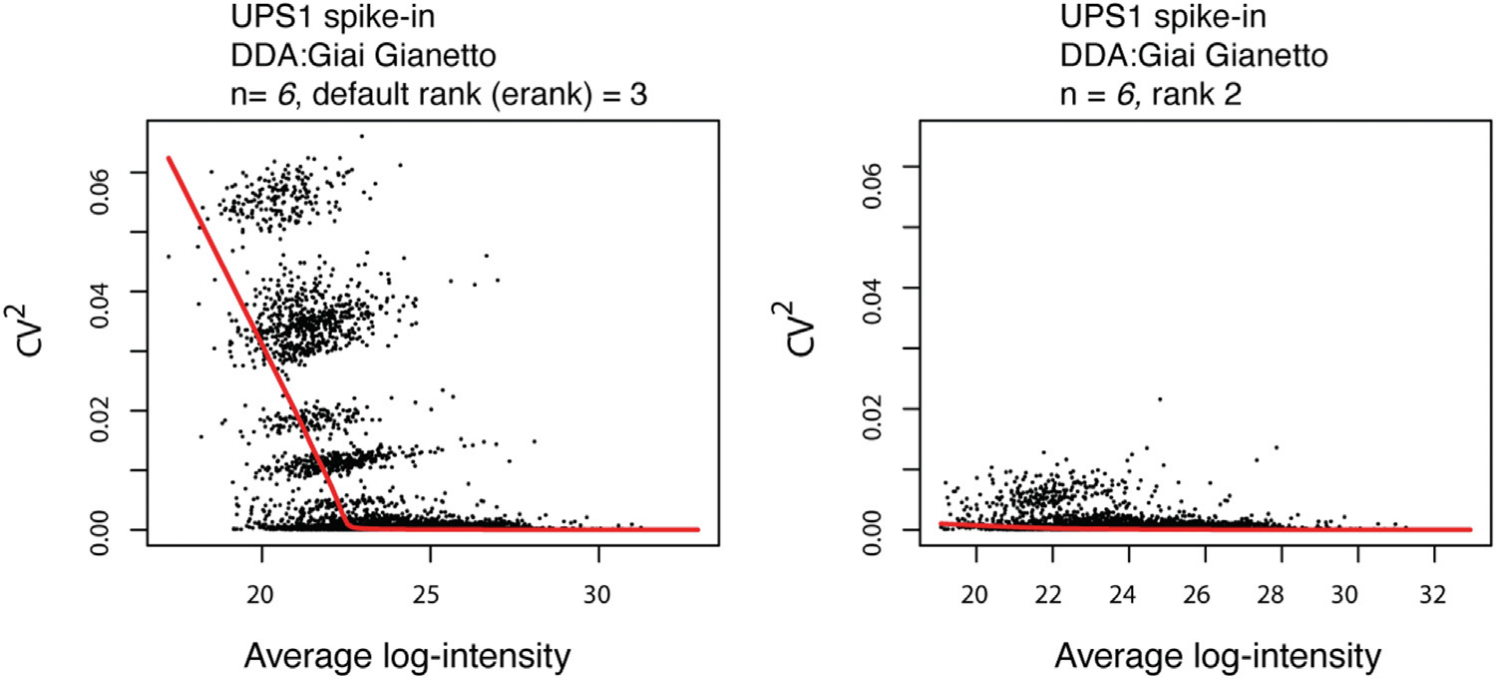
**Comparison between data imputed under the effective rank (erank) model, the default model, and a rank-2 model fitted to the same dataset.** Each point is a peptide. The red curve is a Loess trend fitted on average log intensity and squared coefficient (CV) of variation of the peptide. The presence of the bands under the erank model indicates groups of peptides exhibit similar squared CV and mean abundance, suggesting that the imputation has introduced biases. Rank-2 models provide better approximations in experiments with small (<20) LC-MS runs.

During these evaluations, we also found that for experiments with less than 20 LC-MS runs, rank-2 models perform better than *erank* models, where the rank is estimated by effective rank, for reconstruction of peptide abundance under the MAR assumption. For example, in the DDA UPS1 spike-in dataset by Gianetto *et al.* (29), estimation of rank by effective rank introduces additional variability, measured by the coefficient of variation (CV), in the data at the lower range of the abundance (Fig. 4). However, rank-2 reconstruction of the data appropriately reconstructs the variability for low-abundance peptides with more reasonable coefficient of variation (here CV is squared to improve visualization). In general, one would expect that imputation should not over-smooth the data, resulting in a lower CV than that of original data after imputation. Also, one would not expect dramatic increases in CV and the introduction of undesired variability in the data after imputation. The mean-CV plots in Figure 4 can serve as informative diagnostic plots, in addition to exploratory plots such as Multi-Dimensional Scaling (MDS) and Principal Component Analysis (PCA) plots, to assess the impact of imputation on variability in the data.

### Barycenter Interpolation Reduces False Positives in Differential Abundance Analyses

**Fig. 5:**
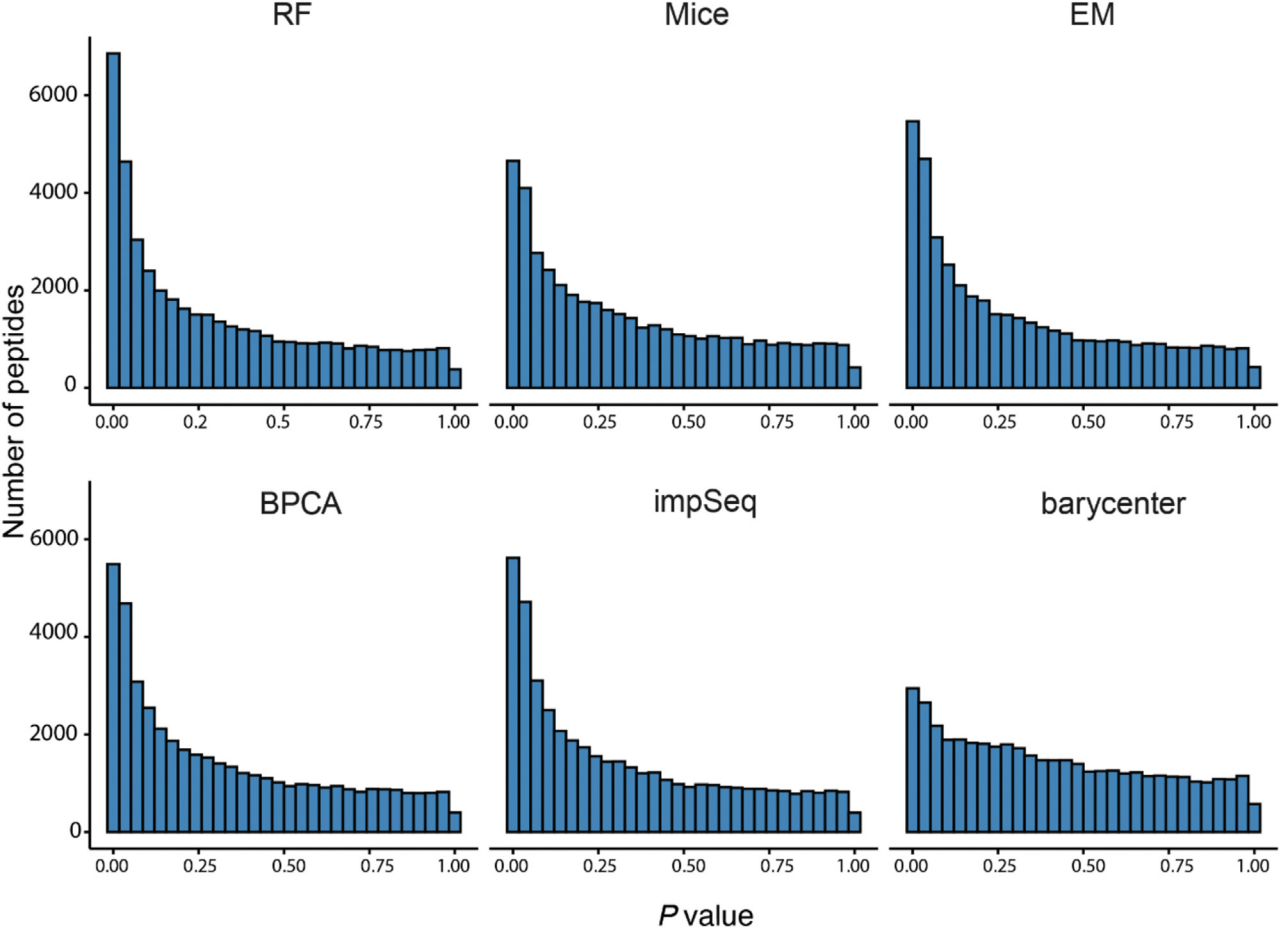
**Distribution of *p* values under the Null hypothesis in ten DDA-PASEF HeLa replicates.** The closer the distribution to uniformity the better.

We used the ten HeLa cell lysate replicates by Prianichnikov *et al.* (35) to assess false discoveries introduced by imputation in differential abundance analyses under the Null model (no biological difference). The ten HeLa cell lysates are technical replicates and contain no biological differences. Therefore, we expect a uniform *p*-value distribution if the replicates are randomly assigned to two groups and a 2-group differential abundance test is performed, as none of the peptides would be DA between the two groups. This analysis was designed to substitute simulation studies, where the attempt is to generate a distribution of abundance values under the Null model; that is - in the absence of biological variability. Protein abundance is assumed to follow a Normal distribution (20) and is commonly simulated from this distribution (9, 10, 41) taking into account the intensity-dependent occurrence of missing values. However, additional variations will be introduced into protein abundance measurements by the quantification software, for example, due to peptide misidentification during match-between-runs, which would not be necessarily captured in simulated measurements sampled from Gaussian models. To address this, we used real technical replicates to provide a fairer representation of variability in the abundance measurements under the Null model. Overall, the distribution of *p*-values departed from uniformity for all the imputation algorithms (Fig. 5). However, the departure was less for the barycenter approach, suggesting that this method results in smaller false-positive DA calls. For the rest of the imputation algorithms, a large proportion of false rejections (of the null hypothesis) was apparent by the spikes in the frequency of *p*-values that were <0.05, suggesting that imputation has introduced false positives in the results. This was a consistent observation from ROC curves (Fig. 2), where the barycenter approach was found to maintain the largest area under the curve in most benchmark datasets that contain real biological variability.

### Barycenter Interpolation is Applicable to Major Quantification Pipelines and Data Processing Tools

We additionally investigated if the choice of the processing tool impacts the imputation outcome of the barycenter approach. We retrieved quantifications of a DDA-controlled mixture dataset by Chiva *et al.* (2014) obtained by MaxQuant, Progenesis, Skyline, and Proteome Discoverer from the MassIVE.quant resource. We studied the sensitivity and specificity of the barycenter approach in data quantified by these processing workflows in the context of differential abundance analysis in supplemental Fig. S2*C*. We observed that the ROC curves are indistinguishable at 1% and 5% FDR (supplemental Fig. S2*C*-i). Overall, we observed that the barycenter approach performs as well in data processed by Progenesis as MaxQuant quantifications. The apparent difference between performance in MaxQuant and Progenesis datasets, and Proteome Discoverer and Skyline datasets can be explained by difference in the proportion of missing values reported by the different processing tools; that is if we stratify the False-Positive, True Positive, and False-Negative DA calls by imputed and non-imputed peptides, the performance is comparable in datasets with similar rates of missing values (supplemental Fig. S2*C*-ii). These results collectively indicate that the barycenter approach is not biased toward a processing tool.

## Discussion

The msImpute software and barycenter imputation algorithm are aware of both the MAR and MNAR nature of missing values in DDA and DIA data acquisition workflows. MsImpute uses entropy-based metrics to quantify the likeliness of each of MAR and MNAR assumptions for a peptide with missing values. Under the MAR assumption, low-rank models are used to reconstruct peptide abundance measurements. Under the MNAR assumption, missing values are estimated by the down-shift approach tailored for the imputation of left-censored MNAR data. The missing values are finally estimated as a weighted mean of the distribution of peptide abundance measurements reconstructed under these assumptions. An entropic metric is used to weigh the MAR and MNAR reconstructions based on the evidence in the data. The current implementation of msImpute uses EBM to distinguish MAR and MNAR MV types and uses fixed weights to compute the barycenter of the two distributions. In our study on the effect of different choices of the weight assigned to each distribution, we did not observe dramatic performance changes. We, therefore, speculate that the barycenter approach should stay reliable for different choices of the weight parameter, and the ranking of DA calls should not be substantially impacted by an incorrect specification of this parameter. It should be noted that the msImpute package comes with a reasonable number of diagnostic plots and metrics, for example, the distribution of EBM values as a function of average abundance as presented in violin plots or those in supplemental Fig. S3. We encourage the user to use their judgment of the data to determine if values other than the ones specified in the original formulation should be used.

In six differential abundance tasks carried on published controlled mixture and UPS1/2 standard proteins spiked into constant and heterogeneous backgrounds, the barycenter algorithm outperformed or had comparable performance to the state-of-the-art, general-purpose imputation algorithms. Additionally, in a HeLa cell lysate dataset with ten replicates where no biological variability was present between the LC-MS runs, the barycenter approach resulted in a smallest false differential abundance (false positive) calls among other algorithms. This can be explained by the underestimation of variance by general-purpose imputation algorithms, which tend to replace missing values with values that are correlated with the observed values, resulting in smaller variance between the measurements, larger test statistics, and correspondingly more rejections of the Null hypothesis when it is true. In contrast, the barycenter approach proposed here is specifically designed for the imputation of MS data, and we demonstrated that it reduces oversmoothing of the variance estimates when compared to the general-purpose algorithms. This in turn results in fewer incorrect rejections of the Null hypothesis and a more uniform *p*-value distribution.

In datasets with few LC-MS runs, we observed that the MAR model of the barycenter approach can introduce undesired variability in peptide abundance measurements. We speculate that the suboptimal reconstruction of variation in peptide abundance measurements by erank approach in the MAR model is due to insufficient sample size (*i.e.*, too few LC-MS runs). We, therefore, recommend rank-2 models for small-scale experiments with less than 20 LC-MS runs. For larger scale experiments, the default rank estimation by the erank approach should appropriately approximate the distribution of missing peptide abundance measurements under MAR assumption. For experiments involving offline fractionation, we recommend merging the runs obtained from a single sample to ensure that the assumptions of the low-rank model are not violated. Note that although 2-group designs were considered in the DA benchmark analyses covered here, the method is applicable to complex experimental designs (*e.g.*, time-series, multiple conditions, combination of groups and time course, etc.) if the experimental groups of interest can be discretized. For example, a time-series dataset with three time points on control and treated samples can be formulated as control_T1, treated_T1, control_T2, treated_T2, and so on.

Although imputation makes common data analysis tasks such as clustering, classification, differential abundance, and pathway enrichment analysis practical by means of enhancing data completeness, it can obscure the amount of available information, particularly if imputed values are considered as equally certain as the observed values in downstream processing. In the benchmark analyses presented here, we observed that empirical FDR exceeds the nominal 5% FDR in DA analysis, suggesting that better statistical models or procedures are required to model DA of imputed peptides. A possible explanation is that the uncertainty of imputed measurements is never accounted for when using the standard limma linear model. The DA models and procedures described by Ahlmann-Eltze & Anders (20) and Zhu *et al.* (42) may improve empirical FDR, however, these methods are not applicable to imputed data. Therefore, procedures are required to accompany imputed values with statistical confidence estimates. These confidence estimates can be used to fit weighted least square estimates instead of the ordinary least square estimates using existing frameworks in limma to improve differential abundance results. The design of such statistical confidence estimate procedures is a future direction of our work.

## Data Availability

All the datasets analyzed in this manuscript are public and published in other papers. We have referenced them in the manuscript. The figures in this manuscript are available in the notebooks containing the code that produced the results of this paper. The notebooks can be downloaded *via* https://github.com/DavisLaboratory/msImpute-reproducibility.

## Code Availability

MsImpute is a R/Bioconductor package and is available for download here https://bioconductor.org/packages/release/bioc/html/msImpute.html.

For latest developments, readers are also encouraged to access the R package from https://github.com/DavisLaboratory/msImpute.

## Supplemental data

This article contains supplemental data.

## Conflict of interest

The authors declare no competing interests.
