# Supplementary material for: MsImpute: Estimation of Missing Peptide Intensity Data in Label-Free Quantitative Mass Spectrometry
Source: Mol Cell Proteomics. 2023 Apr 25;22(8):100558. doi: 10.1016/j.mcpro.2023.100558 (PMC10368900; doi:10.1016/j.mcpro.2023.100558)
Supplement: Supplemental Figures S1–S3 [file mmc1.pdf]

Supplementary Figures

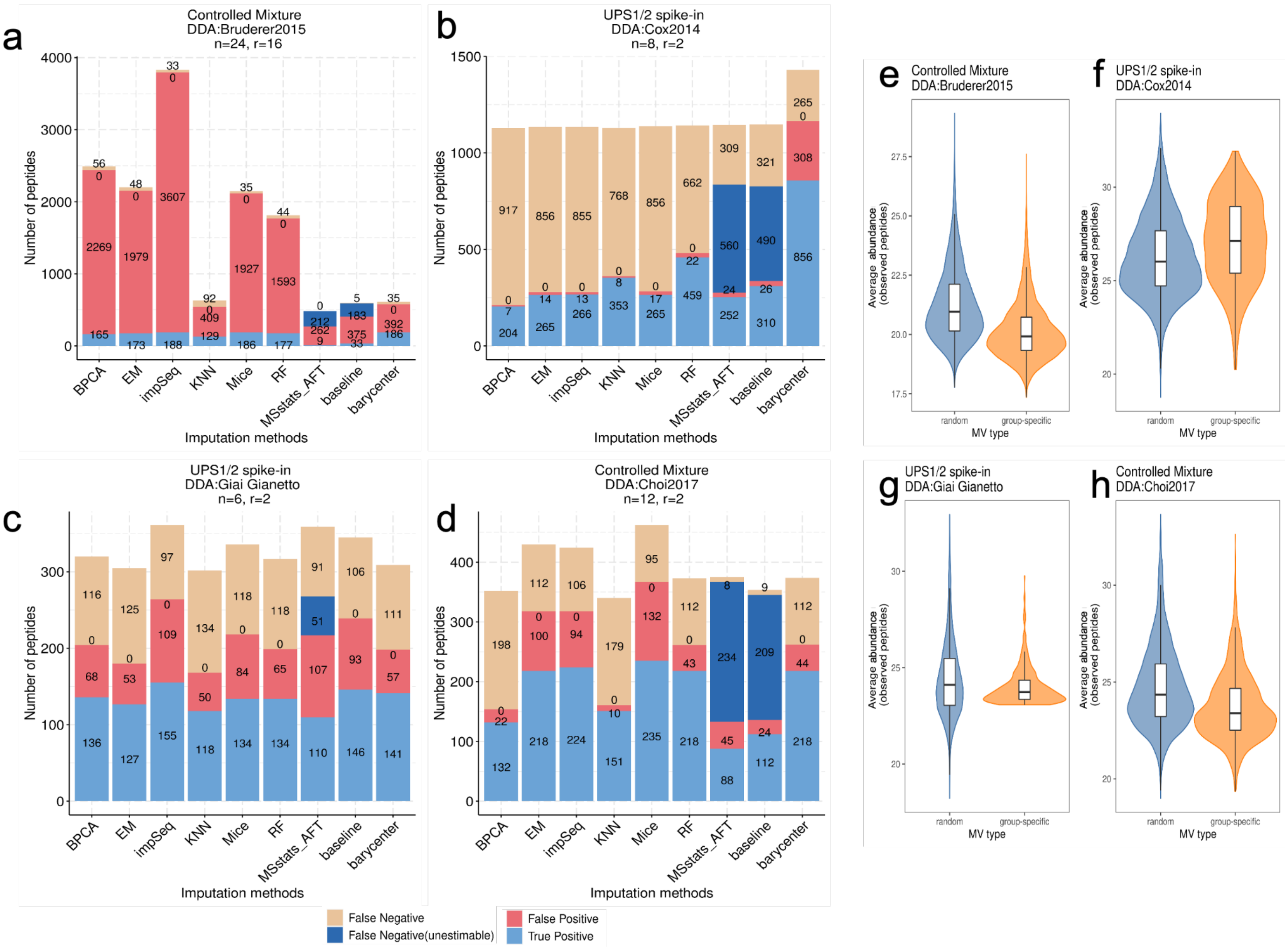

Supplementary Figure S1: **(a-d)** The number of True Positives, False Positives and False Negative peptides in differential abundance test results by imputation method. The test is performed at 5% FDR. **(e-h)** Violin plot of average abundance of peptides exhibiting group-specific and random missing types. The color here represents the type of the missing values for the peptides: The Orange color denotes group-specific missing, that is MNAR type, and the blue color indicates MAR type. The type of missing values is determined by the EBM metric.

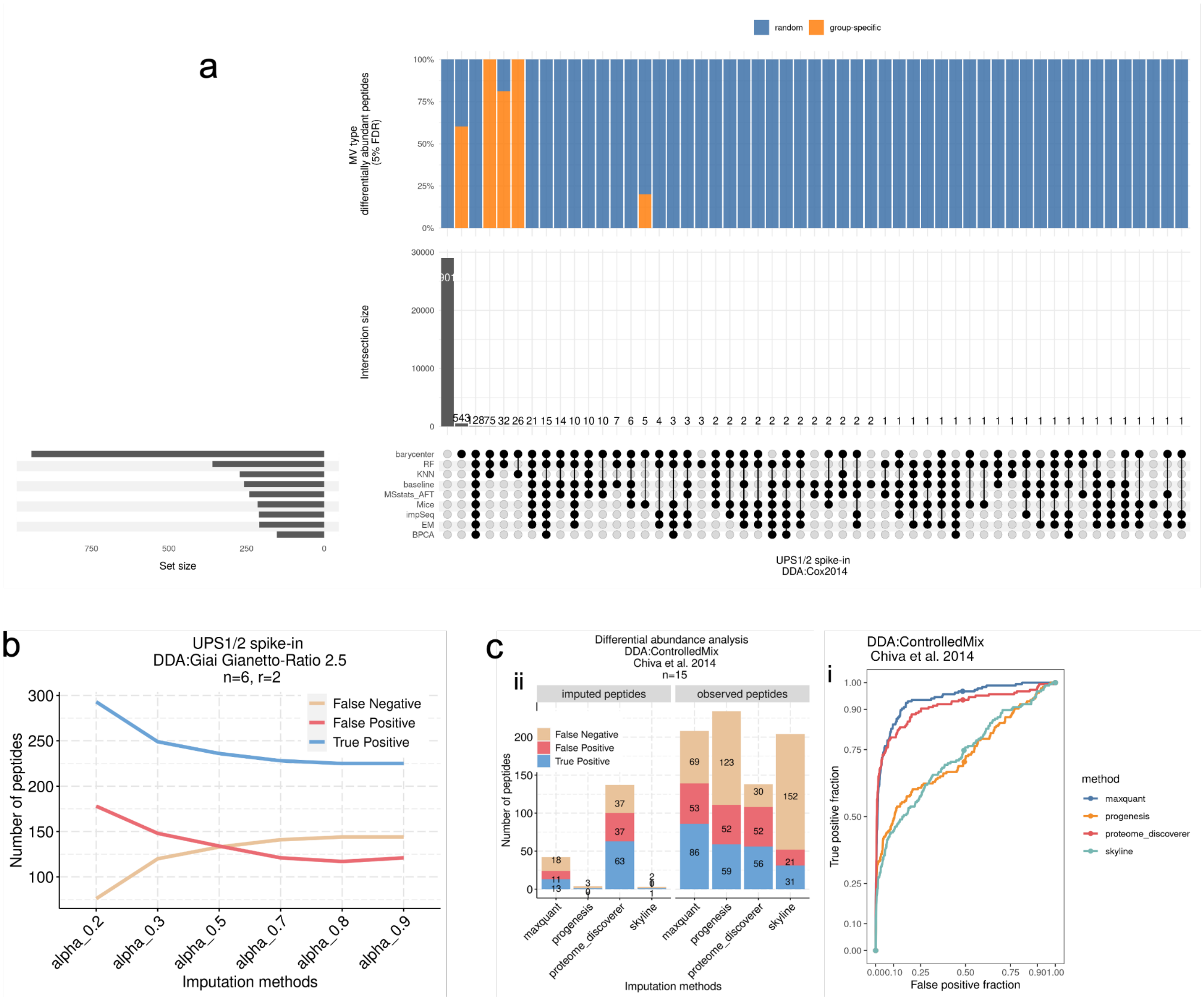

Supplementary Figure S2: (a) Overlap of differential abundant (DA) peptides between imputation methods in DDA:Cox2014 UPS1/2 spike-in dataset. Each row is DA results by a method, and each column is an overlap of DA calls between the methods. The set overlaps are indicated by black dots. There are 543 DA peptides unique to barycenter (second column from left). More than 50% of those are group-specific, suggesting that the barycenter approach has identified group-specific MNAR peptides not detected by other imputation procedures. (b) The impact of the choice of alpha parameter (the weight assigned to MAR distribution in barycenter computation) on True Positive, False Positive and False Negative outcomes in the DA analysis. Low False Negative, high True Positive, and low False Positives are desired. (c) Assessment of the impact of quantification workflow on the performance of the barycenter approach (i) ROC of DA calls in a DDA controlled mixture dataset quantified by MaxQuant, Progenesis, Proteome Discoverer and skyline. The apparent difference in performance of the imputation between data processed by MaxQuant and Progensis, and Proteome Discoverer and Skyline is explained by the proportion of missing peptides in the quantification. When DA calls are stratified based on whether or not the peptide intensity was imputed, we observe similar performance of barycenter in various quantification workflows, suggesting that barycenter approach is not biased towards a specific quantification workflow.

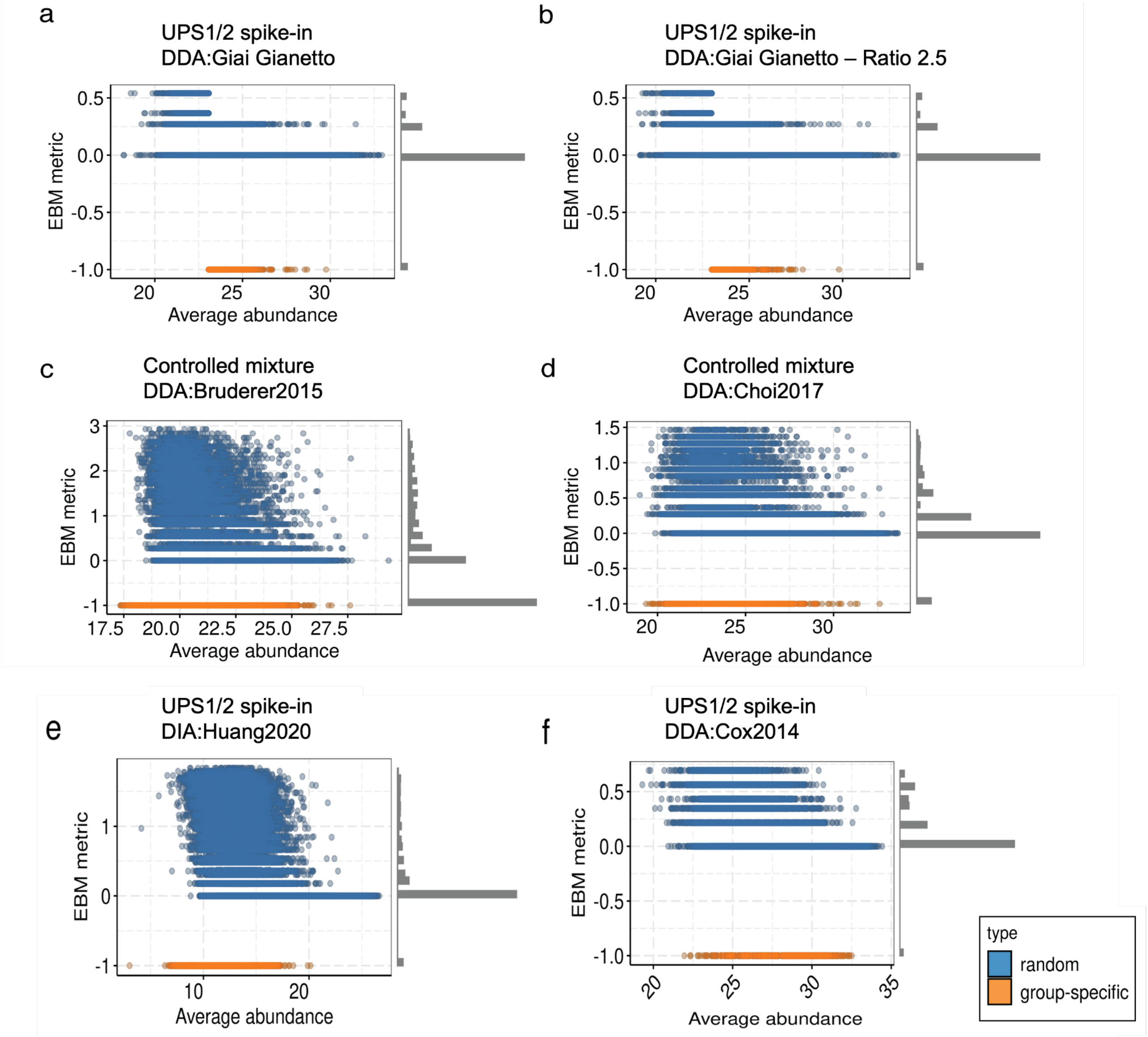

Supplementary Figure S3: The EBM metric (y-axis) and mean abundance (x-axis) of peptides in each of the benchmark datasets. Each point represents a peptide. The secondary y-axis is a histogram of the number of peptides with a specific EBM value. The color denotes the type of missing values for a given peptide. Blue represents peptide missing at random, i.e., MAR type, and orange represents group-specific missing or MNAR type. The average abundance is determined as the average of observed log peptide intensity before imputation.
